# Human Leukocyte Antigen Class I and Class II Polymorphisms and Serum Cytokine Profiles in Cervical Cancer

**DOI:** 10.3390/ijms18091478

**Published:** 2017-08-31

**Authors:** Larissa Bahls, Roger Yamakawa, Karina Zanão, Daniela Alfieri, Tamires Flauzino, Francieli Delongui, André de Abreu, Raquel Souza, Fabrícia Gimenes, Edna Reiche, Sueli Borelli, Marcia Consolaro

**Affiliations:** 1 Laboratory of Immunogenetics, Department of Basic Health Sciences, State University of Maringá (UEM), 87020-900 Maringá, PR, Brazil; laribahls@gmail.com (L.B.); spruem2011@gmail.com (R.Y.); karinazanao6@gmail.com (K.Z.) sueliborelli@gmail.com (S.B.); 2 Laboratory of Clinical Cytology, Department of Clinical Analysis and Biomedicine, State University of Maringá (UEM), 87020-900 Maringá, PR, Brazil; andreluelsdorf@gmail.com (A.d.A); raquelpantarotto@gmail.com (R.S.); fabricia.gimenes@gmail.com (F.G.); 3 Post Graduate Program of Biosciences and Physiopathology, Clinical Analysis and Biomedicine Department, State University of Maringá (UEM), 87020-900 Maringá, PR, Brazil; 4 Laboratory of Clinical and Toxicological Analysis, Department of Pathology, State University of Londrina (UEL), 86057-970 Londrina, PR, Brazil; frizon.alfieri@gmail.com (D.A.); t.flauzino@hotmail.com (T.F.); frandelongui@hotmail.com (F.D.); reiche@sercomtel.com.br (E.R.)

**Keywords:** genes, major histocompatibility complex class I, genes, major histocompatibility complex class II, serum cytokines, papillomavirus infections, uterine cervical dysplasia, uterine cervical neoplasms

## Abstract

Only a small proportion of women who are exposed to infection with high-risk human papillomavirus (HR-HPV) progress to persistent infection and develop cervical cancer (CC). The immune response and genetic background of the host may affect the risk of progression from a HR-HPV infection to lesions and cancer. However, to our knowledge, no studies has been conducted to evaluate the relationship between variability of human leukocyte antigens (*HLA*) genes and serum cytokine expression in this pathology. In the current study, we examined the associations of *HLA* alleles and haplotypes including Class I (*HLA-A*, *-B* and *-C*) and II (*HLA-DRB1*, *-DQA1* and *-DQB1*) with serum levels of cytokines interleukin (IL)-6, tumor necrosis factor-α (TNF-α), IL-10 and IL-17 as well as risks of HPV infections, lesions and CC among admixed Brazilian women. HLA polymorphisms were associated with an increased risk or protection from HPV, lesions and CC. Additionally, we demonstrated a potential association of a *HLA* class I haplotype (HLA-B*14-C*08) with higher IL-10 cytokine serum levels in cervical disease, suggesting an association between HLA class I and specific cytokines in cervical carcinogenesis. However, larger studies with detailed HPV types coupled with genetic data are needed to further evaluate the effects of HLA and CC by HPV genotype.

## 1. Introduction

Cervical cancer (CC) is currently the fourth leading cause of cancer mortality in women worldwide, despite the existence of highly effective prevention and screening methods [1]. It is already established that persistent infection with high-risk human papillomavirus (HR-HPV) is necessary for CC development [2]. Genital HPV infection is very common among female population in general. However, only a small fraction of these women progress to persistent HPV infection and thus, have an increased risk of developing cervical lesions and invasive CC [3,4]. Growing evidence suggests that long-term HPV infection is necessary but not enough to induce carcinogenesis [2,3,4,5]. In this regard, the host immune response and its genetic background may be among the most significant contributing factors in the natural history of HR-HPV-associated diseases [6,7].

The activation of an effective immune response against virus-infected and tumor cells involve the antigen presentation to specific T cells. Human leukocyte antigens (HLA) play an important role in this process by selecting peptides generated from the processing of foreign antigens and exposing them on the surface of the cell for T cell recognition [8]. HLA class I molecules are expressed by nucleated cells and present intracellular antigens to cytotoxic T cells, while HLA class II molecules are present on the surface of professional antigen-presenting cells (dendritic cells, macrophages and B lymphocytes) and also in some tumor cells. These class II molecules present extracellular antigens to helper T cells [9]. Genes encoding HLA molecules are highly polymorphic and variability between individuals determines the different peptides that are presented to T lymphocytes. Consequently, HLA diversity possibly determines different ability of immune-recognition and clearance of HPV infection, which subsequently creates different susceptibility to neoplastic progression [10,11,12,13,14,15,16,17]. However, technical and financial limitations have restricted studies to a low number of participants, with previous studies on HLA class I and II polymorphisms and susceptibility to CC having obtained mixed results in different populations [16].

Depending on the stimulus of activation, T-helper lymphocytes may differentiate with at least three subtypes, each one producing their own pattern of cytokines: T-helper 1 (Th1), T-helper 2 (Th2) or T-helper 17 (Th17). Cytokines are known to have an important function in defending against HPV infection, regulating viral transcription and modulating viral replication [18,19,20,21]. Classically, Th1 cytokines, such as interleukin-6 (IL-6), IL-12, tumor necrosis factor-α (TNF-α), and interferon-γ (IFN-γ), promote immune responses to intracellular pathogens and tumor cells. Th2 cytokines, such as IL-4, IL-5, IL-10 and IL-13, mainly induce immune responses to helminths and parasites. In comparison, Th17 cytokines, such as IL-17 and IL-22, stimulate immune protection against extracellular bacteria/fungi, while its role in tumor immunity remains controversial [22,23]. However, increased levels of Th1 cytokines, including TNF-α and IL-6, have been associated with persistent HPV infection, CC and metastasis [24,25,26]. Additionally, other cytokines related to immune response modulation, such as IL-17 and IL-10, appears to play a paradoxical role in cervical disease, although mixed results have been reported [19,26]. Despite it being known that the HLA/peptide complex generated after antigen immunization dictates which cytokine profile production is preferentially induced [27], no studies has been conducted to evaluate the relationship between variability of *HLA* genes and serum cytokine expression in the HR-HPV cervical infections to our knowledge.

In the current study, we examined the associations of *HLA* alleles and haplotypes, including *HLA* Class I (*HLA-A*, *-B* and -*C*) and Class II (*HLA-DRB1*, *-DQA1* and *-DQB1*), with serum levels of cytokines IL-6, TNF-α, IL-10 and IL-17 as well as the risk of HPV infections, cervical lesions and invasive CC among Southern Brazilian women of highly admixed descent of a geographic area with a high incidence of CC. Our main finding was higher IL-10 serum levels in women carrying the HLA-B*14-C*08 haplotype, which is related to protection against HR-HPV infection and severe cervical lesions/CC. This suggests a possible association between HLA class I and specific cytokines in cervical carcinogenesis.

## 2. Results

The mean ages of the 124 women included in the study were similar among the different groups used for statistical analyses. The average ages were as follows: 33.29 years for women with a negative histological diagnosis for intraepithelial lesions or malignancy (NILM); 31.04 years for women with low-grade squamous intraepithelial lesions (LSIL); and 36.1 years for women with high-grade squamous intraepithelial lesions (HSIL) or CC.

A total of 39 HPV genotypes were detected: 17 HR-HPV, 21 low-risk HPV (LR-HPV) and 1 undetermined-risk HPV (UR-HPV). Of the 124 women included, 78 (62.9%) were HPV-positive. Of these HPV-positive women, 16 (20.5%) had only LR-HPV, 48 (61.5%) had only HR-HPV, while14 (18.0%) had multiple HPV infections with LR-HPV, HR-HPV and/or UR-HPV genotypes. HPV-16 was the most prevalent in the overall population (*n* = 29, 31.2%), followed by HPV-18 (*n* = 6, 7.7%), HPV-51 and HPV-82 (*n* = 5, 7.4% for both), HPV-33 and HPV-45 (*n* = 4, 5.9%) in addition to others (frequency lower than 5%). Table 1 shows the prevalence of total HPV, HR-HPV and HPV-16 by different cytological findings, in which genotypes of multiple HPV infections were counted several times.

For *HLA* class I, 16 *HLA-A*, 25 *HLA-B* and 14 *HLA-C* different allelic groups were detected. For *HLA* class II, 13 *HLA-DRB1*, 6 *HLA-DQA1* and 6 *HLA-DQB1* different allelic groups were detected. The most frequent allelic groups in the study population were HLA-A*02 (*n* = 69, 27.8%), HLA-B*44 (*n* = 29, 11.7%), HLA-C*07 (*n* = 68, 27.4%), HLA-DRB1*07 (*n* = 33, 13.3%), HLA-DQA1*01 (*n* = 89, 35.9%) and HLA-DQB1*03 (*n* = 73, 29.4%) (Table 2). The *HLA* allelic distribution showed a Hardy–Weinberg equilibrium for all loci studied, except for the *DQB1* locus.

The frequencies of *HLA* allelic groups and haplotypes were compared, with HPV positivity and different cytological findings examined to detect possible associations. Associations of *HLA* allelic groups and HPV infections or LSIL, HSIL and CC that were significant before Bonferroni correction were grouped as shown in Table 3. HLA-B*07 (3.26% versus 11.54%, *p* = 0.0316, OR = 3.87, CI = 1.11–13.52) and HLA-DQB1*03 (20.83% versus 34.87%, *p* = 0.0265, OR = 2.03, CI = 1.12–3.69) allelic groups were more frequently found in women with HPV infections and LSIL/HSIL/CC, respectively. In contrast, the frequency of HLA-B*14 and HLA-C*08 allelic groups was decreased in women who had HR-HPV infections and LSIL/HSIL/CC (6.35% versus 0.82%, *p =* 0.0359, OR *=* 0.12, CI *=* 0.15–0.99; and 6.25% versus 0.0%, *p* = 0.0271, respectively for both allelic groups).

Statistically significant associations of *HLA* haplotypes and HPV infections were grouped as shown in Table 4. The following *HLA* haplotypes were associated with increased risk: HLA-B*15-C*03-DQB1*03 for HPV infections (0.0% versus 7.8%, *p* = 0.0065, *p*_c_ = 0.0325); HLA-A*02-B*40 and HLA-A*03-DQB1*06 for HR-HPV infections (0.0% versus 7.3%, *p* = 0.0029, *p*_c_ = 0.0202; and 0.0% versus 7.8%, *p* = 0.0029, *p*_c_ = 0.0289, respectively); and HLA-A*02-C*03 and HLA-A*03-DQB1*06 for HPV-16 infections (2.1% versus 7.1%, *p* = 0.0072, *p*_c_ = 0.0362, OR = 4.18, CI = 1.53–11.39; and 1.7% versus 11.6%, *p* = 0.0037, *p*_c_ = 0.0409, OR = 8.56, CI = 2.14–34.26, respectively).

In contrast, the following *HLA* haplotypes were associated with decreased risk (protection): A*11-B*35-C*04-DRB1*01-DQA1*01-DQB1*05 (6.2% versus 0.0%, *p* = 0.0047, *p*_c_ = 0.0094), A*11-B*35-C*04-DQB1*05 (6.2% versus 0.0%, *p* = 0.0047, *p*_c_ = 0.0141), A*11-B*35-C*04 (6.2% versus 0.0%, *p* = 0.0047, *p*_c_ = 0.0281), A*11-B*35-DQB1*05 (6.2% versus 0.0%, *p* = 0.0047, *p*_c_ = 0.0141), and A*11-C*04-DQB1*05 (6.2% versus 0.0%, *p* = 0.0047, *p*_c_ = 0.0328) for HPV infections; HLA-A*01-B*08-C*07-DQB1*02 and B*08-C*07-DQB1*02 for HR-HPV infections (6.3% versus 0.0%, *p* = 0.0079; *p*_c_ = 0.0397; and 7.9% versus 0.0%, *p* = 0.0019; *p*_c_ = 0.0115, respectively); and HLA-A*01-B*08-C*07-DQB1*02 also for HPV-16 infections (5.1% versus 0.0%, *p* = 0.0019; *p*_c_ = 0.0076) (Table 4).

Statistically significant associations of *HLA* haplotypes with LSIL and HSIL/CC were also grouped, as shown in Table 4. The following *HLA* haplotypes were associated with increased risk: HLA-B*15-C*03-DQB1*03 (0.0% versus 8.1%, *p* = 0.0073, *p*_c_ = 0.0439) and C*03-DQB1*03 (0.0% versus 8.1%, *p* = 0.0004, *p*_c_ = 0.005) for LSIL and HSIL/CC. In contrast, the following *HLA* haplotypes were associated with decreased risk (protection): A*11-B*35-C*04-DRB1*01-DQA1*01-DQB1*05 (5.9% versus 0.0%, *p* = 0.0061, *p*_c_ = 0.0122), A*11-B*35-C*04-DQB1*05 (5.9% versus 0.0%, *p* = 0.0061, *p*_c_ = 0.0183), A*11-B*35-C*04 (5.9% versus 0.0%, *p* = 0.0061, *p*_c_ = 0.0488), A*11-B*35-DQB1*05 (6.3% versus 0.0%, *p* = 0.0061, *p*_c_ = 0.0244) and A*11-C*04-DQB1*05 (6.3% versus 0.0%, *p* = 0.0061, *p*_c_ = 0.0366) for LSIL and HSIL/CC.

**Figure 1:**
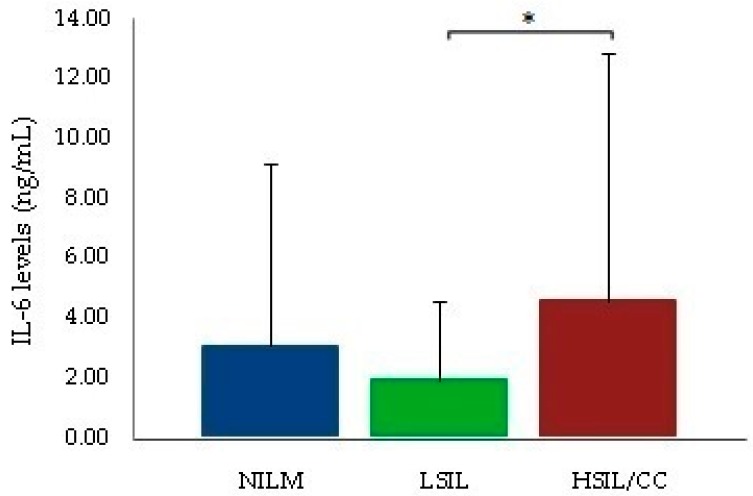
Comparison of mean IL-6 levels between women with LSIL and HSIL/CC. Note that IL-6 = interleukin-6. * 1.96 ± 2.62 versus 4.59 ± 8.27; *p* = 0.046.

**Figure 2:**
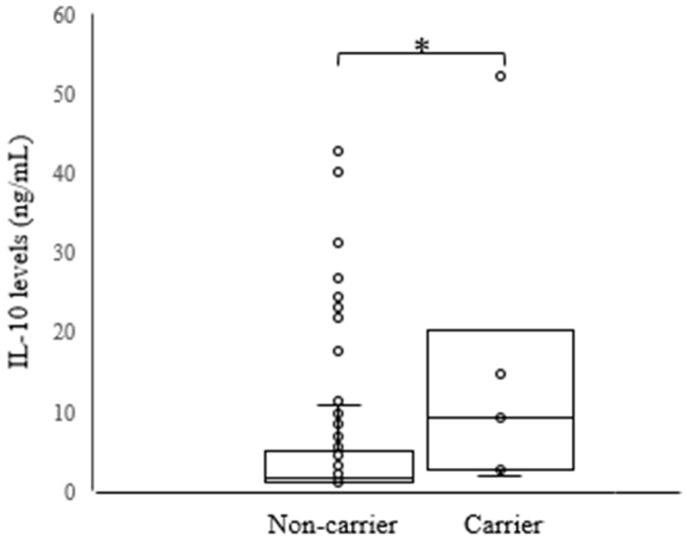
Comparison of mean IL-10 levels between haplotype HLA-B*14-C*08 carrier and non-carrier women, regardless of cytological findings and HPV status. Note that IL-10 = interleukin-10. * 5.47 ± 8.68 versus 14.87 ± 17.82; *p* = 0.021.

With respect to the cytokine profile, IL-6 serum levels were higher in women with HSIL/CC than in those with LSIL (4.59 ± 8.27 versus 1.96 ± 2.62, *p* = 0.0459) (Figure 1). Furthermore, TNF-α, IL-10 and IL-17 serum levels were not statistically associated with HPV positivity or with any different cytological findings. However, higher IL-10 serum levels were associated with the HLA-B*14-C*08 haplotype (5.47 ± 8.68 versus 14.87 ± 17.82, *p* = 0.0209) regardless of cytological findings and HPV status (Figure 2). The HLA-B*14-C*08 haplotype was associated with protection against HR-HPV infections (6.35% versus 0.82%, *p* = 0.0402, *p*_c_ = 0.2813) and against HSIL/CC (6.25% versus 0.0%, *p* = 0.0271, *p*_c_ = 0.1895) as previously described.

## 3. Discussion

In the present study, we analyzed the associations of *HLA* alleles and haplotypes, including *HLA* Class I (*HLA-A*, *-B*, *-C*) and *HLA* Class II (*HLA-DRB1*, *-DQA1* and *-DQB1*), with serum levels of cytokines IL-6, TNF-α, IL-10 and IL-17 as well as the risk of HPV infections, cervical lesions and invasive CC among Brazilian women of highly admixed descent. To our knowledge, this is the first study to demonstrate a potential association between *HLA* polymorphisms and serum cytokine levels. Our main finding was an association of higher IL-10 serum levels with the HLA-B*14-C*08 haplotype, which showed a protective effect for HR-HPV infections and for HSIL/CC. Additionally, higher IL-6 serum levels were associated with HSIL/CC. Finally, the HLA-B*07 and HLA-DQB1*03 allelic groups as well as the HLA-B*15-C*03-DQB1*03, HLA-A*02-B*40, HLA-A*02-C*03 and HLA-A*03-DQB1*06 haplotypes were associated with increased risk of HPV infection and/or for LSIL/HSIL/CC. In contrast, the HLA-B*14 and HLA-C*08 allelic groups as well as the HLA-A*11-B*35-C*04-DRB1*01-DQA1*01-DQB1*05 and HLA-A*01-B*08-C*07-DQB1*02 haplotypes were associated with protection against HPV infections and LSIL/HSIL/CC. However, considering that the total number of samples included in the study was limited in order to minimize the risk of population–stratification bias, larger studies with detailed HPV types coupled with high quality *HLA* data are needed to further evaluate the effects of *HLA* and CC by HPV genotype.

HPV is necessary but not sufficient to cause CC, which supports the hypothesis of an influence of additional factors in carcinogenesis associated with HR-HPV [28]. An association between *HLA* genes and cervical carcinogenesis has been hypothesized due to the high degree of polymorphism of *HLA* genes, which confers different abilities of immune-recognition and clearance of infections. Thus, this would result in different degrees of susceptibility to HPV and neoplastic progression [10,11,12,13,14,15,16,17]. An effective immune response may require optimal peptide presentation by both HLA class I and class II molecules to activate HPV specific cytotoxic and helper T cells, respectively. Subtle changes in or impairment of T cell responses may allow escape from immune surveillance, induction of immune anergy or tolerance to HPV peptides [19]. Our results are consistent with this hypothesis, since we showed that some *HLA* alleles and haplotypes were associated with an increased risk of or protection against HPV, cervical lesions and cancer. Additionally, we demonstrated a potential association of *HLA* class I polymorphism with higher IL-10 cytokine levels in cervical disease.

The HLA-B*14:02 and HLA-C*08:02 alleles were previously associated with an increased risk of LSIL compared to HSIL or CC [17]. Our data was consistent with this result, although significance was lost after Bonferroni correction. A decreased frequency of HR-HPV infections and severe cervical abnormalities (HSIL and CC) was observed in women carrying HLA-B*14 and HLA-C*08 alleles or the HLA-B*14-C*08 haplotype. Reinforcing our evidence of protective effects of these *HLA* alleles and haplotype, HLA-B*14-C*08 was potentially associated with higher serum levels of IL-10, independently of HPV infection or cervical abnormalities status. IL-10 is known as an important regulator of the immune system, which attenuates exaggerated immune responses that can lead to deleterious tissue lesions by inhibiting the production of proinflammatory cytokines by both antigen-presenting cells (APCs) and CD4^+^ T cells [29,30]. IL-10 has both immunosuppressive and antiangiogenic effects. Therefore, this interleukin may exert both tumor-promoting and antitumor effects [29,30,31,32]. Associations with both increased and decreased IL-10 levels in CC have been shown in different studies [30]. Additionally, IL-10 was previously related to increased proliferation and cytotoxic function of HPV-specific CD8^+^ T cells as well as with increased expression of IFN-γ [31]. Thus, it is possible that higher levels of IL-10 in the women carrying the HLA-B*14-C*08 haplotype may be involved in the clearance of HPV infections by stimulating cytotoxic T cells (CTLs) against HPV-infected cells. Furthermore, the protection from cervical disease progression may be due to the anti-inflammatory and anti-angiogenic effects of IL-10. However, more studies involving cytokine polymorphisms are needed to clarify the link between IL-10 and HLA in order to assess this hypothesis.

In relation to other cytokines studied, IL-6 serum levels were significantly higher in women with HSIL/CC than in those with LSIL. These findings are in agreement with other studies in which IL-6 promoted CC growth by inhibiting apoptosis and increasing neo-angiogenesis [27,33,34].

Our results revealed an association between some allelic groups and haplotypes with an increased risk of HPV infections and/or cervical lesions and CC. All associations found for allelic groups were previously described by others, as discussed below. Thus, although significance was lost after Bonferroni correction for allelic groups, we believe these associations may be considered as being possibly valid. The HLA-B*07 allelic group was more frequent in HPV infected women, with this same allelic group associated with increased risk of HPV infection in other reports [11,13,22,35,36]. The HLA-DQB1*03 allelic group and the HLA-B*15-C*03-DQB1*03 haplotype was associated with increased risk of HPV infections and of LSIL and HSIL/CC. This finding is in agreement with others that demonstrated the association of HLA-DQB1*03 alleles with increased risk of CC [15,37,38,39,40,41]. Furthermore, the HLA-A*02-B*40 and HLA-A*02-C*03 haplotypes were associated with an increased risk of HPV and HPV-16 infections, respectively. Previous studies reported conflicting results in relation to an association of HLA-A*02 alleles with increased risk of [35] or protection from [42,43] CC and HPV-16 infections. Finally, the HLA-A*03-DQB1*06 haplotype was associated with increased risk of both HPV and HPV-16 infections, as described previously for the DQB1*06 allele [44,45,46].

In contrast, we showed an association of allelic groups (although significance was lost after Bonferroni correction) and haplotypes with protection from HPV infections and/or lesions and CC. Specifically, the HLA-B*14 and HLA-C*08 allelic groups in addition to the HLA-A*11-B*35-C*04-DRB1*01-DQA1*01-DQB1*05 haplotype were associated with protection from HPV infections, lesions and CC. Curiously, only the isolated alleles HLA-C*04, DRB1*01, DQA1*01 and DQB1*05:01 were previously associated with a protective effect [17,35,39,41,47,48,49], but not the HLA-A*11-B*35-C*04-DRB1*01-DQA1*01-DQB1*05 haplotype. Nevertheless, we showed that the HLA-A*01-B*08-C*07-DQB1*02 haplotype was associated with a protective effect, specifically against HPV-16 infections. Otherwise, the alleles DQB1*02:01 and DQB1*02:01/02 were associated with a higher risk of HPV-16 infection [50] and of CC [51], respectively.

Some limitations in our study should be noted. First, we have no data on the history of HPV infections and progression of cervical lesions, which limits our interpretations of the influence of *HLA* polymorphisms and serum cytokines on HPV persistence. Second, cases of women matching the same ethnic background and residing in the same geographical areas were eligible, in order to minimize the risk of population–stratification bias. As a consequence, a total number of participants included in the study was limited. However, it is reassuring that the frequency of *HLA* alleles among the women in our study is consistent with findings from other reports from the same state [52] and also from other admixed populations [49]. Furthermore, since HPV variants have coevolved with different ethnic groups, the host genetic factors may influence the viral variants, so developing a study with a population that has a highly admixed ethnic background is advantageous [53]. It is well known that HPV types 16 and 18 are the most frequent types worldwide, with HPV-16 the most common type in all regions [54,55]. Similar trends were observed in the present study, with HPV-16 being the most prevalent. Nevertheless, considering that HPV-16 is responsible globally for 60.6% of CC worldwide [55,56], further studies with restricted analysis of HPV-16 may enable a more robust analysis of HLA type-specific peptides.

## 4. Materials and Methods

### 4.1. Study Population

A total of 265 unrelated admixed Brazilian women from the state of Paraná in southern Brazil were recruited for the study. All women were referred for colposcopy due to previous abnormal cytological findings (Pap smears) at the colposcopy clinic of the Public Health Hospital in the city of União da Vitória from October 2012 to September 2014.

According to Probst et al. [57], the population of Paraná as a whole is predominantly of European origin, with a smaller but significant African and Amerindian contribution. Therefore, the study population was considered to be admixed. Considering these characteristics, women were selected by matching the same ethnic background and residing in the same geographical areas, to minimize the risk of population–stratification bias. Women were excluded due to: previous history of squamous intraepithelial lesions or cervical, vaginal or vulvar cancer; pregnancy; postpartum; previous hysterectomy; previous history of other cancers; without history of sexual activity; recent treatment for any pathology of the urogenital tract; ablative or excisional therapy to the cervix within the previous 12 months; chronic diseases (diabetes, allergy or autoimmune disease) and immunocompromised state. Additionally, samples of 10 women were excluded due to low quality of the extracted blood DNA, even after the extraction process was repeated.

Considering all the criteria adopted, 124 women formed the study population, with an age range from 18 to 67 years and a mean age and standard deviation (SD) of 40.3 ± 10.0 years. The study subjects included: (1) 27 women who underwent colposcopy due to LSIL in referral Pap smears, who had a confirmed histologic diagnosis of cervical intraepithelial neoplasia (CIN) grade I; (2) 42 women who underwent colposcopy due to atypical squamous cells (ASC) but not possible to exclude HSIL (ASC-H) or high-grade SIL (HSIL) in referral Pap smears, who had a confirmed histologic diagnosis of CIN II, CIN III or in situ carcinoma; (3) 7 women who underwent colposcopy due to squamous-cell cervical carcinoma (SCC) in referral Pap smears, who had a confirmed histologic diagnosis of invasive CC; and (4) 48 women who underwent colposcopy due to ASC of undetermined significance (ASC-US) in referral Pap smears, who had a negative histologic diagnosis of intraepithelial lesion CIN or malignancy (NILM).

All participants voluntarily agreed to provide biological samples for HPV detection, HLA typing and cytokine determination. They all gave written informed consent before enrollment. This study was approved by the Committee for Ethics in Research Involving Humans at the State University of Maringá (UEM), Brazil, and registered in the National Commission for Research Ethics (CONEP), Ministry of Health of Brazil (No. 132.503/2012, 24 September 2012).

### 4.2. Sample Collection

In all patients, colposcopy was performed after application of 3% acetic acid. Colposcopically targeted biopsies were taken from the most abnormal area of the cervix. An endocervical curettage was performed if the transformation zone was not entirely visible. Women with a suspicious image penetrating the cervical canal and those in whom colposcopy was unsatisfactory were submitted to cervical conization. The material was fixed in formaldehyde. The morphological diagnosis of cervical biopsies was assessed according to the International Histological Classification of Tumors [58].

According to the Brazilian Cervical Cancer Guidelines, all cases with cytological findings of ASC-H, HSIL and CC should be analyzed by colposcopy and histology, before being properly treated and/or having a follow-up [59]. At colposcopy, cervical samples were collected with a cytobrush, immediately suspended in 1 ml of sterile 0.9% NaCl solution and frozen at −80 °C until analyzed. For HLA typing and cytokine serum determination, blood samples were collected using Ethylenediamine tetraacetic acid (EDTA) tubes and serum tubes, respectively. Buffy-coat was obtained from EDTA tubes after centrifugation at 1500 rpm for 10 min. Following this first processing, samples were immediately stored at −20 °C until use.

### 4.3. DNA Extraction and HPV Genotyping

An AxyPrep™ Body Fluid Viral DNA/RNA Miniprep Kit (Axygen Scientific, Union City, CA, USA) was used for DNA extraction according to the manufacturer’s instructions. The quality and concentration of purified DNA were measured by spectrophotometry (NanoDrop 2000 Spectrophotometer, Thermo Scientific, Wilmington, DE, USA).

A single-target polymerase chain reaction (PCR) method has been in use in our laboratory for several years for HPV detection, which consists of HPV-PCR amplification carried out using primers MY09 (5′-CGTCCMAARGGAWACTGATC-3′) and MY11 (5′-GCMCAGGGWCATAAYAATGG-3′). For the reaction, 2.5 mM of each deoxynucleotide (dNTP), 1 U of Taq DNA polymerase (Invitrogen, Carlsbad, CA, USA), 0.6 mM of MgCl_2_, 25 mM of each primer and 50 ng of extracted DNA for a final volume of 15 μL was used. Co-amplification of the human β-globin gene was performed as an internal control, using primers GH20 (5′-GAAGAGCCAAGGACAGGTAC-3′) and PC04 (5′-CAACTTCATCCACGTTCACC-3′) under the same conditions as the HPV-PCR. Two types of controls were also included in each reaction: “no-DNA” (negative control) and “HPV-positive DNA” (positive control). PCR products were electrophoresed in 1.0% agarose gel, stained with 1.0 μg/mL ethidium bromide and photo-documented under UV light.

HPV-positive samples were genotyped by PCR-RFLP (Restriction Fragment Length Polymorphism), as described previously [60]. For RFLP, 10 μL of each sample was digested in a final volume of 15 μL with the restriction enzyme HpyCH4V (New England Biolabs, Ipswich, MA, USA) according to the manufacturer’s instructions. Restriction fragments were resolved in 8% polyacrylamide gels. HPV genotypes were determined by analyzing each band with Labimage 1D software (Loccus Biotechnology, São Paulo, Brazil), and the molecular weights were compared for HPV genotype determination. A total of 39 individual HPV-DNA genotypes—17 genotypes considered to be either high-risk or potentially high-risk, 22 low-risk genotypes not associated with carcinogenesis and 1 genotype with undetermined risk of carcinogenesis—can be determined by the PCR-RFLP method. In the present study, the following genotypes were considered high-risk: 16, 18, 31, 33, 35, 39, 45, 51, 52, 53, 56, 58, 59, 66, 68, 73 and 82 [61].

### 4.4. HLA Class I and II Genotyping

Genomic DNA from blood samples was extracted using a Mini Spin Plus Extraction Kit (Biometrix Diagnóstica, Paraná, Brazil) according to the manufacturer’s instructions. The quality and quantity of purified DNA were measured using a NanoDrop 2000 Spectrophotometer (Thermo Scientific).

As instructed by the LABType^®^ SSO (One Lambda, Inc., Canoga Park, CA, USA) manufacturer, the purified DNA obtained from blood samples was used to amplify locus *HLA* Class I (*HLA-A*, *-B*, *-C*) and Class II (*HLA-DRB1*, *-DQA1* and *-DQB1*), before the amplification products were hybridized to sequence-specific oligonucleotide probes (SSO) immobilized on fluorescently coded microspheres. After the hybridization, the samples were read using a LABScan™ 100 flow cytometer (One Lambda, Inc.) and the results were analyzed in HLA Fusion software version 3.0 (One Lambda, Inc.) to identify possible *HLA* alleles.

### 4.5. Serum Cytokine Measurements

Serum levels of Th1 (IL-6 and TNF-α), Th2 (IL-10) and Th17 (IL-17) cytokines were measured by a sandwich enzyme-linked immunosorbent assay (ELISA, eBioscience, San Diego, CA, USA) and the results were expressed in ng/mL. The lower limit of detection for these cytokines was defined according to the manufacturer’s instructions (IL-6: 1.0 ng/mL, TNF-α: 2.0 ng/mL, IL-4: 1.0 ng/mL, IL-10: 1.0 ng/mL and IL-17: 2.0 ng/mL).

### 4.6. Statistical Analyses

Different variables/groups were evaluated, as follows: (1) Cytological findings: normal (NILM) and abnormal cytology, grouped into LSIL and HSIL/CC (these two most-severe grades were grouped together); and (2) HPV infections: HPV-positive versus HPV-negative, HR-HPV-positive versus HR-HPV-negative and HPV-16-positive versus HPV-16-negative. Allele and haplotype frequencies were calculated using the Arlequin Program v3.1 (available at: http://cmpg.unibe.ch/software/arlequin3). Alleles and haplotypes present in ≥5% of the women were included in the main analyses. To limit the number of comparisons in the haplotype analyses, only the multiloci combinations of greatest interest was explored. OpenEpi v.3.01 software (www.openepi.com) [62] was used to compare *HLA* allele and haplotype frequencies in different groups, with a two-tailed Fisher’s Exact Test or Yates’ corrected chi square. The Odds Ratio (OR) and 95% Confidence Interval (CI) were calculated in all significant comparisons. A Bonferroni correction was used to avoid a type I error.

Mean levels of cytokine were compared between different groups by Student’s *t*-test for independent samples, using the BioEstat v5.0 program (www.mamiraua.org.br/pt-br/downloads/programas/) [63]. To analyze possible associations between *HLA* polymorphism and the serum cytokine profile (IL-6, TNF-α, IL-10 and IL-17 levels) in carriers and non-carriers of each allelic and haplotype, groups were compared with Kruskal–Wallis analysis of variance or Mann–Whitney test. All *p*-values less than 0.05 were considered statistically significant.

## 5. Conclusions

To our knowledge, this is the first study to demonstrate a potential association of higher IL-10 serum levels with the HLA-B*14-C*08 haplotype, which showed a protective effect for HR-HPV infections and for HSIL/CC. Further investigations involving other polymorphisms with longer-term follow-ups and larger sample sizes will be needed in order to determine whether these findings can be repeated in other studies with different populations. Continued examination of the immune response to HPV may lead to the development of novel therapies or effective vaccines against established HPV infections.

## Figures and Tables

**Table 1:** Frequency of HPV infection grouped by cytological findings.

Cytology	Women	HPV Positivity		
		HPV-DNA	HR-HPV	HPV-16
	*n* (%)	*n* (%)	*n* (%)	*n* (%)
NILM	48 (38.7)	3 (6.3)	2 (66.7)	0 (0.0)
LSIL	27 (21.8)	26 (96.3)	12 (46.2)	06 (23.1)
HSIL	42 (33.9)	42 (100.0)	41 (97.6)	19 (45.2)
CC	07 (5.6)	07 (100.0)	07 (100.0)	04 (57.1)
Total	124 (100.0)	78 (62.9)	62 (79.5)	29 (37.2)

HPV = human papillomavirus; NILM = negative for intraepithelial lesion or malignancy; LSIL = low-grade squamous intraepithelial lesion; HSIL = high-grade squamous intraepithelial lesion; and CC = cervical cancer.

**Table 2:** *HLA* class I and II allele types in the study population (*n* = 124).

*HLA-A*	*n* (*f*%)	B*40	17 (6.9)	*HLA-DRB1*	*n* (*f*%)
A*01	28 (11.3)	B*41	1 (0.4)	DRB1*01	31 (12.5)
A*02	69 (27.8)	B*42	1 (0.4)	DRB1*03	26 (10.5)
A*03	21 (8.5)	B*44	29 (11.7)	DRB1*04	24 (9.7)
A*11	18 (7.3)	B*45	3 (1.2)	DRB1*07	33 (13.3)
A*23	9 (3.6)	B*48	4 (1.6)	DRB1*08	27 (10.9)
A*24	28 (11.3)	B*49	4 (1.6)	DRB1*09	6 (2.4)
A*25	3 (1.2)	B*50	4 (1.6)	DRB1*10	3 (1.2)
A*26	9 (3.6)	B*51	16 (6.5)	DRB1*11	21 (8.5)
A*29	14 (5.7)	B*52	7 (2.8)	DRB1*12	3 (1.2)
A*30	3 (1.2)	B*55	2 (0.8)	DRB1*13	24 (9.7)
A*31	24 (9.7)	B*56	4 (1.6)	DRB1*14	10 (4.0)
A*32	8 (3.2)	B*57	6 (2.4)	DRB1*15	28 (11.3)
A*33	2 (0.8)	B*58	5 (2.0)	DRB1*16	12 (4.8)
A*66	3 (1.2)	*HLA-C*	**n (f%)**	*HLA-DQA1*	**n (f%)**
A*68	7 (2.8)	C*01	10 (4.0)	DQA1*01	89 (35.9)
A*74	2 (0.8)	C*02	13 (5.2)	DQA1*02	34 (13.7)
*HLA-B*	**n (f%)**	C*03	40 (16.1)	DQA1*03	31 (12.5)
B*07	21 (8.5)	C*04	39 (15.7)	DQA1*04	26 (10.5)
B*08	22 (8.9)	C*05	11 (4.4)	DQA1*05	67 (27.0)
B*13	4 (1.6)	C*06	17 (6.9)	DQA1*06	1 (0.4)
B*14	9 (3.6)	C*07	68 (27.4)	*HLA-DQB1*	**n (f%)**
B*15	28 (11.3)	C*08	9 (3.6)	DQB1*01	2 (0.8)
B*18	10 (4.0)	C*12	13 (5.2)	DQB1*02	55(22.2)
B*27	6 (2.4)	C*14	1 (0.4)	DQB1*03	73 (29.4)
B*35	28 (11.3)	C*15	10 (4.0)	DQB1*04	21 (8.5)
B*37	2 (0.8)	C*16	12 (4.8)	DQB1*05	49 (19.8)
B*38	5 (2.0)	C*17	3 (1.2)	DQB1*06	48 (19.4)
B*39	10 (4.0)	C*18	2 (0.8)		

*HLA* = human histocompatibility antigen.

**Table 3:** Associations of *HLA* allelic groups with risk of HPV infection or cervical lesions/cancer development.

*HLA* Allelic Group	n (ƒ%)	n (ƒ%)	*p*-Value	OR CI (95%)
	HPV-negative (*n* = 46)	HPV-positive (*n* = 78)		
B*07	3 (3.26)	18 (11.54)	0.0316 ^1^	3.87 (1.11–13.52)
	HR-HPV-negative *(n* = 63)	HR-HPV-positive (*n* = 61)		
B*14	8 (6.35)	1 (0.82)	0.0359 ^1^	0.12 (0.15–0.99)
C*08	8 (6.35)	1 (0.82)	0.0359 ^1^	0.12 (0.15–0.99)
	NILM (*n* = 48)	HSIL/CC (*n* = 49)		
B*14	6 (6.25)	0 (0.0)	0.0271 ^1^	-
C*08	6 (6.25)	0 (0.0)	0.0271 ^1^	-
DQB1*03	20 (20.83)	35 (35.71)	0.0324 ^2^	2.11 (1.11–4.02)
	NILM (*n* = 48)	LSIL/HSIL/CC (*n* = 76)		
DQB1*03	20 (20.83)	53 (34.87)	0.0265 ^2^	2.03 (1.12–3.69)

^1^ *p*-Value calculated with Fisher’s exact test; ^2^ *p*-value calculated with Yates’ corrected chi square. All comparisons had *p*_c_ (corrected by Bonferroni test) > 0.05. Note that *p*_c_ = *p*-value corrected by Bonferroni test; OR = odds ratio; and CI = confidence interval.

**Table 4:** Significant associations of *HLA* haplotypes with risk of HPV infection or cervical lesions/cancer development.

*HLA* Haplotypes	HPV		HR-HPV		HPV-16		NILM (*n* = 48)	LSIL (*n* = 27)	HSIL/CC (*n* = 49)
	Yes (*n* = 78)	No (*n* = 46)	Yes (*n* = 61)	No (*n* = 63)	Yes (*n* = 29)	No (*n* = 95)			
	*n* (*f*%) *	*n* (*f*%) *	*n* *(f*%) *	*n* (*f*%) *	*n* (*f*%) *	*n* (*f*%) *	*n* (*f*%) *	*n* (*f*%) ^†^	*n* (*f*%) ^†^
A*11-B*35-C*04-DRB1*01-DQA1*01-DQB1*05	0 (0.0) ^a^	6 (6.2) ^a^	0 (0.0)	6 (4.8)	0 (0.0)	6 (3.0)	6 (5.9) ^b^	0 (0.0) ^b^	0 (0.0) ^b^
A*11-B*35-C*04-DQB1*05	0 (0.0) ^a^	6 (6.2) ^a^	0 (0.0)	6 (4.8)	0 (0.0)	6 (3.0)	6 (5.9) ^b^	0 (0.0) ^b^	0 (0.0) ^b^
A*11-B*35-C*04	0 (0.0) ^a^	6 (6.2) ^a^	0 (0.0)	7 (5.4)	0 (0.0)	7 (3.5)	6 (5.9) ^b^	0 (0.0) ^b^	0 (0.0) ^b^
A*11-B*35-DQB1*05	0 (0.0) ^a^	6 (6.2) ^a^	0 (0.0)	6 (4.8)	0 (0.0)	6 (3.0)	6 (6.3) ^b^	0 (0.0) ^b^	0 (0.0) ^b^
A*11-C *04-DQB1*05	0 (0.0) ^a^	6 (6.2) ^a^	0 (0.0)	6 (4.8)	0 (0.0)	6 (3.0)	6 (6.3) ^b^	0 (0.0) ^b^	0 (0.0) ^b^
A*01-B*08-C*07-DQB1*02	0 (0.0)	4 (4.3)	0 (0.0) ^c^	8 (6.3) ^c^	0 (0.0) ^d^	10 (5.1) ^d^	4 (4.2)	0 (0.0)	1 (1.0)
B 08-C*07-DQB1*02	10 (6.4)	4 (4.3)	0 (0.0) ^c^	10 (7.9) ^c^	0 (0.0)	13 (6.8)	4 (4.2)	5 (9.2)	5 (5.1)
B*15-C*03-DQB1*03	12 (7.8) ^a^	0 (0.0) ^a^	9 (7.4)	5 (4.0)	4 (6.9)	10 (5.1)	0 (0.0) ^b,e^	4 (7.4) ^b^	8 (8.1) ^b,e^
C*03-DQB1*03	16 (10.0) ^a^	0 (0.0) ^a^	12 (9.9)	3 (2.7)	6 (9.9)	10 (5.4)	0 (0.0) ^b,e^	3 (5.6) ^b^	12 (12.7) ^b,e^
A*02-B*40	9 (5.8)	0 (0.0)	9 (7.3) ^c^	0 (0.0) ^c^	5 (8.6)	4 (2.1)	0 (0.0)	0 (0.0)	8 (7.9)
A*02-C*03	11 (7.1)	2 (2.1)	11 (9.0)	3 (2.3)	9 (15.5) ^d,†^	8 (4.4) ^d,‡^	2 (1.9)	2 (3.7)	11 (11.2)
A*03-DQB1*06	9 (5.7)	2 (2.2)	9 (7.8) ^c^	0 (0.0) ^c^	7 (11.6) ^d,‡^	3 (1.7) ^d,‡^	2 (2.1)	4 (7.4)	5 (4.7)
B*14-C*08	3 (1.9)	6 (6.5)	1 (0.8) ^c^	8 (6.4) ^c^	0 (0.0)	9 (4.7)	6 (6.3) ^e^	3 (5.6)	0 (0.0) ^e^

* Haplotype frequency calculated using Arlequin software. All comparisons had *p* values < 0.01 and *p*_c_ (corrected by Bonferroni test) < 0.05, except B*14-C*08; ^a^ HPV-negative versus HPV-positive; ^b^ NILM versus LSIL/HSIL/CC; ^c^ HR-HPV-negative versus HR-HPV-positive; ^d^ HPV-16-negative versus HPV-16-positive; ^e^ NILM versus HSIL/CC; ^†^ OR (CI 95%) = 4.18 (1.53–11.39); ^‡^ OR (CI 95%) = 8.56 (2.14–34.26); Note that HR-HPV = high-risk HPV.

## Abbreviations

CC	Cervical cancer
HPV	Human papillomavirus
HR-HPV	High-risk human papillomavirus
HLA	Human leukocyte antigens
Th1	T-helper 1
Th2	T-helper 2
Th17	T-helper 17
IL-6	Interleukin 6
IL-12	Interleukin 12
TNF-α	Tumor necrosis factor-α
IFN-γ	Interferon-γ
IL-4	Interleukin-4
IL-5	Interleukin-5
IL-10	Interleukin-10
IL-13	Interleukin-13
IL-17	Interleukin-17
IL-22	Interleukin-22
NILM	Negative for intraepithelial lesion or malignancy
LSIL	Low-grade squamous intraepithelial lesions
HSIL	High-grade squamous intraepithelial lesions
OR	Odds ratio
CI	Confidence interval
CTLs	Cytotoxic T cells
SD	Standard deviation
CIN	Cervical intraepithelial neoplasia
ASC	Atypical squamous cells
ASC-H	ASC but not possible to exclude HSIL
ASC-US	ASC of undetermined significance
PCR	Polymerase chain reaction
RFLP	Restriction fragment length polymorphism
SSO	Sequence-specific oligonucleotide
ELISA	Enzyme-linked immunosorbent assay

## References

[1] Ferlay J., Soerjomataram I., Dikshit R., Eser S., Mathers C., Rebelo M., Parkin D.M., Forman D., Bray F. (2015). Cancer incidence and mortality worldwide: Sources, methods and major patterns in GLOBOCAN 2012. Int. J. Cancer.

[2] Walboomers J.M., Jacobs M.V., Manos M.M., Bosch F.X., Kummer J.A., Shah K.V., Snijders P.J., Peto J., Meijer C.J., Muñoz N. (1999). Human papillomavirus is a necessary cause of invasive cervical cancer worldwide. J. Pathol..

[3] Rodríguez A.C., Schiffman M., Herrero R., Wacholder S., Hildesheim A., Castle P.E., Solomon D., Burk R. (2008). Rapid clearance of human papillomavirus and implications for clinical focus on persistent infections. J. Natl. Cancer Inst..

[4] Franco E.L., Villa L.L., Sobrinho J.P., Prado J.M., Rousseau M.C., Désy M., Rohan T.E. (1999). Epidemiology of acquisition and clearance of cervical human papillomavirus infection in women from a high-risk area for cervical cancer. J. Infect. Dis..

[5] Ho G.Y., Bierman R., Beardsley L., Chang C.J., Burk R.D. (1998). Natural history of cervicovaginal papillomavirus infection in young women. N. Engl. J. Med..

[6] Tota J.E., Chevarie-Davis M., Richardson L.A., Devries M., Franco E.L. (2011). Epidemiology and burden of HPV infection and related diseases: Implications for prevention strategies. Prev. Med..

[7] Hong J.H., Kim M.K., Lee I.H., Kim T.J., Kwak S.H., Song S.H., Lee J.K. (2010). Association between serum cytokine profiles and clearance or persistence of high-risk human papillomavirus infection: A prospective study. Int. J. Gynecol. Cancer.

[8] Gimenes F., Teixeira J.J., de Abreu A.L., Souza R.P., Pereira M.W., da Silva V.R., Bôer C.G., Maria-Engler S.S., Bonini M.G., Borelli S.D. (2014). Human leukocyte antigen (HLA)-G and cervical cancer immunoediting: A candidate molecule for therapeutic intervention and prognostic biomarker?. Biochim. Biophys. Acta.

[9] Hildesheim A., Wang S.S. (2002). Host and viral genetics and risk of cervical cancer: A review. Virus Res..

[10] Wu T.C. (1994). Immunology of the human papilloma virus in relation to cancer. Curr. Opin. Immunol..

[11] Chen D., Juko-Pecirep I., Hammer J., Ivansson E., Enroth S., Gustavsson I., Feuk L., Magnusson P.K., McKay J.D., Wilander E. (2013). Genome-wide association study of susceptibility loci for cervical cancer. J. Natl. Cancer Inst..

[12] de Araujo Souza P.S., Sichero L., Maciag P.C. (2009). HPV variants and HLA polymorphisms: The role of variability on the risk of cervical cancer. Future Oncol..

[13] Hildesheim A., Schiffman M., Scott D.R., Marti D., Kissner T., Sherman M.E., Glass A.G., Manos M.M., Lorincz A.T., Kurman R.J. (1998). Human leukocyte antigen class I/II alleles and development of human papillomavirus-related cervical neoplasia: Results from a case-control study conducted in the United States. Cancer Epidemiol. Biomark. Prev..

[14] Madeleine M.M., Brumback B., Cushing-Haugen K.L., Schwartz S.M., Daling J.R., Smith A.G., Nelson J.L., Porter P., Shera K.A., McDougall J.K. (2002). Human leukocyte antigen class II and cervical cancer risk: A population-based study. J. Infect. Dis..

[15] Madeleine M.M., Johnson L.G., Smith A.G., Hansen J.A., Nisperos B.B., Li S., Zhao L.P., Daling J.R., Schwartz S.M., Galloway D.A. (2008). Comprehensive analysis of HLA-A, HLA-B, HLA-C, HLA-DRB1, and HLA-DQB1 loci and squamous cell cervical cancer risk. Cancer Res..

[16] Shi Y., Li L., Hu Z., Li S., Wang S., Liu J., Wu C., He L., Zhou J., Li Z. (2013). A genome-wide association study identifies two new cervical cancer susceptibility loci at 4q12 and 17q12. Nat. Genet..

[17] Wang S.S., Hildesheim A., Gao X., Schiffman M., Herrero R., Bratti M.C., Sherman M.E., Barnes W.A., Greenberg M.D., McGowan L. (2002). Comprehensive analysis of human leukocyte antigen class I alleles and cervical neoplasia in 3 epidemiologic studies. J. Infect. Dis..

[18] Wang S.S., Hildesheim A., Gao X., Schiffman M., Herrero R., Bratti M.C., Sherman M.E., Barnes W.A., Greenberg M.D., McGowan L. (2002). Human leukocyte antigen class I alleles and cervical neoplasia: No heterozygote advantage. Cancer Epidemiol. Biomark. Prev..

[19] Zhu J., Yamane H., Paul W.E. (2010). Differentiation of effector CD4 T cell populations. Annu. Rev. Immunol..

[20] Punt S., Fleuren G.J., Kritikou E., Lubberts E., Trimbos J.B., Jordanova E.S., Gorter A. (2015). Angels and demons: Th17 cells represent a beneficial response, while neutrophil IL-17 is associated with poor prognosis in squamous cervical cancer. Oncoimmunol.

[21] Zijlmans H.J.M.A.A., Punt S., Fleuren G.J., Trimbos J.B., Kenter G.G., Gorter A. (2012). Role of IL-12p40 in cervical carcinoma. Br. J. Cancer.

[22] Wang S.S., Wheeler C.M., Hildesheim A., Schiffman M., Herrero R., Bratti M.C., Sherman M.E., Alfaro M., Hutchinson M.L., Morales J. (2001). Human leukocyte antigen class I and II alleles and risk of cervical neoplasia: Results from a population-based study in Costa Rica. J. Infect. Dis..

[23] Klein J., Sato A. (2000). The HLA system. First of two parts. N. Engl. J. Med..

[24] Mota F., Rayment N., Chong S., Singer A., Chain B. (1999). The antigen-presenting environment in normal and human papillomavirus (HPV)-related premalignant cervical epithelium. Clin. Exp. Immunol..

[25] zur Hausen H. (2002). Papillomaviruses and cancer: From basic studies to clinical application. Nat. Rev. Cancer.

[26] Bailey S.R., Nelson M.H., Himes R.A., Li Z., Mehrotra S., Paulos C.M. (2014). Th17 cells in cancer: The ultimate identity crisis. Front. Immunol..

[27] Paradkar P.H., Joshi J.V., Mertia P.N., Agashe S.V., Vaidya R.A. (2014). Role of cytokines in genesis, progression and prognosis of cervical cancer. Asian Pac. J. Cancer Prev..

[28] de Abreu A.L., Malaguti N., Souza R.P., Uchimura N.S., Ferreira É., Pereira M.W., Carvalho M.D., Pelloso S.M., Bonini M.G., Gimenes F. (2016). Association of human papillomavirus, *Neisseria gonorrhoeae* and *Chlamydia trachomatis* co-infections on the risk of high-grade squamous intraepithelial cervical lesion. Am. J. Cancer Res..

[29] Mege J.L., Meghari S., Honstettre A., Capo C., Raoult D. (2006). The two faces of interleukin 10 in human infectious diseases. Lancet Infect. Dis..

[30] Wang Y., Liu X.H., Li Y.H., Li O. (2013). The paradox of IL-10-mediated modulation in cervical cancer. Biomed. Rep..

[31] Santin A.D., Hermonat P.L., Ravaggi A., Bellone S., Pecorelli S., Roman J.J., Parham G.P., Cannon M.J. (2000). Interleukin-10 increases Th1 cytokine production and cytotoxic potential in human papillomavirus-specific CD8(+) cytotoxic T lymphocytes. J. Virol..

[32] Kohno T., Mizukami H., Suzuki M., Saga Y., Takei Y., Shimpo M., Matsushita T., Okada T., Hanazono Y., Kume A. (2003). Interleukin-10-mediated inhibition of angiogenesis and tumor growth in mice bearing VEGF-producing ovarian cancer. Cancer Res..

[33] Castrilli G., Tatone D., Diodoro M.G., Rosini S., Piantelli M., Musiani P. (1997). Interleukin 1alpha and interleukin 6 promote the in vitro growth of both normal and neoplastic human cervical epithelial cells. Br. J. Cancer.

[34] Wei L.H., Kuo M.L., Chen C.A., Chou C.H., Lai K.B., Lee C.N., Hsieh C.Y. (2003). Interleukin-6 promotes cervical tumor growth by VEGF-dependent angiogenesis via a STAT3 pathway. Oncogene.

[35] Davidson E.J., Davidson J.A., Sterling J.C., Baldwin P.J., Kitchener H.C., Stern P.L. (2003). Association between human leukocyte antigen polymorphism and human papillomavirus 16-positive vulval intraepithelial neoplasia in British women. Cancer Res..

[36] Qiu X., Zhang F., Chen D., Azad A.K., Zhang L., Yuan Y., Jiang Z., Liu W., Tan Y., Tao N. (2011). HLA-B*07 is a high risk allele for familial cervical cancer. Asian Pac. J. Cancer Prev..

[37] Chuang L.C., Hu C.Y., Chen H.C., Lin P.J., Lee B., Lin C.Y., Pan M.H., You S.L., Hsieh C.Y., Chen C.J. (2012). Associations of human leukocyte antigen class II genotypes with human papillomavirus 18 infection and cervical intraepithelial neoplasia risk. Cancer.

[38] De Araujo Souza P.S., Villa L.L. (2003). Genetic susceptibility to infection with human papillomavirus and development of cervical cancer in women in Brazil. Mutat. Res..

[39] Ivansson E.L., Magnusson J.J., Magnusson P.K., Erlich H.A., Gyllensten U.B. (2008). MHC loci affecting cervical cancer risk: Distinguishing the effects of *HLA-DQB1* and non-hla genes *TNF*, *LTA*, *TAP1* and *TAP2*. Genes Immun..

[40] Liang J., Xu A., Xie Y., Awonuga A.O., Lin Z. (2008). Some but not all of HLA-II alleles are associated with cervical cancer in Chinese women. Cancer Genet. Cytogenet..

[41] Zhang X., Zhang L., Tian C., Yang L., Wang Z. (2014). Genetic variants and risk of cervical cancer: Epidemiological evidence, meta-analysis and research review. BJOG.

[42] Chan D.P., Cheung T.H., Tam A.O., Cheung J.L., Yim S.F., Lo K.W., Siu N.S., Zhou D.X., Chan P.K. (2005). Risk association between human leukocyte antigen-A allele and high-risk human papillomavirus infection for cervical neoplasia in Chinese women. J. Infect. Dis..

[43] Hosono S., Kawase T., Matsuo K., Watanabe M., Kajiyama H., Hirose K., Suzuki T., Kidokoro K., Ito H., Nakanishi T. (2010). HLA-A alleles and the risk of cervical squamous cell carcinoma in Japanese women. J. Epidemiol..

[44] Chan P.K.S., Cheung J.L.K., Cheung T.-H., Lin C.K., Siu S.-S.N., Yu M.M.Y., Tang J.W., Lo K.W.K., Yim S.-F., Wong Y.F. (2007). HLA-DQB1 polymorphisms and risk of cervical cancer: A case-control study in a southern Chinese population. Gynecol. Oncol..

[45] Wu Y., Chen Y., Li L., Cao Y., Liu Z., Liu B., Du Z., Zhang Y., Chen S., Lin Z. (2006). Polymorphic amino acids at codons 9 and 37 of HLA-DQB1 alleles may confer susceptibility to cervical cancer among Chinese women. Int. J. Cancer.

[46] Beskow A.H., Gyllensten U.B. (2002). Host genetic control of HPV 16 titer in carcinoma in situ of the cervix uteri. Int. J. Cancer.

[47] Cuzick J., Terry G., Ho L., Monaghan J., Lopes A., Clarkson P., Duncan I. (2000). Association between high-risk HPV types, HLA DRB1* and DQB1* alleles and cervical cancer in British women. Br. J. Cancer.

[48] De Araujo Souza P.S., Maciag P.C., Ribeiro K.B., Petzl-Erler M.L., Franco E.L., Villa L.L. (2008). Interaction between polymorphisms of the human leukocyte antigen and HPV-16 variants on the risk of invasive cervical cancer. BMC Cancer.

[49] Maciag P.C., Schlecht N.F., Souza P.S., Franco E.L., Villa L.L., Petzl-Erler M.L. (2000). Major histocompatibility complex class II polymorphisms and risk of cervical cancer and human papillomavirus infection in Brazilian women. Cancer Epidemiol. Biomark. Prev..

[50] Maciag P.C., Schlecht N.F., Souza P.S., Rohan T.E., Franco E.L., Villa L.L. (2002). Polymorphisms of the human leukocyte antigen DRB1 and DQB1 genes and the natural history of human papillomavirus infection. J. Infect. Dis..

[51] Lin P., Koutsky L.A., Critchlow C.W., Apple R.J., Hawes S.E., Hughes J.P., Touré P., Dembele A., Kiviat N.B. (2001). HLA class II DR-DQ and increased risk of cervical cancer among Senegalese women. Cancer Epidemiol. Biomark. Prev..

[52] Marangon A.V., Guelsin G.A., Visentainer J.E., Borelli S.D., Watanabe M.A., Consolaro M.E., Caleffi-Ferracioli K.R., Rudnick C.C., Sell A.M. (2013). The association of the immune response genes to human papillomavirus-related cervical disease in a Brazilian population. BioMed Res. Int..

[53] Wang S.S., Hildesheim A. (2003). Viral and host factors in human papillomavirus persistence and progression. J. Natl. Cancer Inst. Monogr..

[54] Bruni L., Diaz M., Castellsagué X., Ferrer E., Bosch F.X., de Sanjosé S. (2010). Cervical human papillomavirus prevalence in 5 continents: Meta-analysis of 1 million women with normal cytological findings. J. Infect. Dis..

[55] (2017). Human papillomavirus vaccines: WHO position paper, May 2017. Wkly. Epidemiol. Rec..

[56] Serrano B., de Sanjosé S., Tous S., Quiros B., Muñoz N., Bosch X., Alemany L. (2015). Human papillomavirus genotype attribution for HPVs 6, 11, 16, 18, 31, 33, 45, 52 and 58 in female anogenital lesions. Eur. J. Cancer.

[57] Probst C.M., Bompeixe E.P., Pereira N.F., de O’Dalalio M.M., Visentainer J.E., Tsuneto L.T., Petzl-Erler M.L. (2000). HLA polymorphism and evaluation of European, African, and Amerindian contribution to the white and mulatto populations from Paraná, Brazil. Hum. Biol..

[58] Sobin L.H. (1981). The international histological classification of tumours. Bull. World Health Organ..

[59] Katz L.M.C. (2016). Review of the Brazilian guidelines for cervical cancer screening, 2016. J. Bras. Patol. Med. Lab..

[60] Chen L., Watanabe K., Haruyama T., Kobayashi N. (2013). Simple and rapid human papillomavirus genotyping method by restriction fragment length polymorphism analysis with two restriction enzymes. J. Med. Virol..

[61] Santiago E., Camacho L., Junquera M.L., Vázquez F. (2006). Full HPV typing by a single restriction enzyme. J. Clin. Virol..

[62] Sullivan K.M., Dean A., Soe M.M. (2009). Openepi: A web-based epidemiologic and statistical calculator for public health. Public Health Rep..

[63] Ayres M., Ayres Júnior M., Ayres D.L., Santos A.S.D. (2007). Bioestat 5.0: Aplicações Estatísticas nas Áreas das Ciências Biológicas e Médicas.

